# T cell subsets, regulatory T, regulatory B cells and proinflammatory cytokine profile in *Schistosoma haematobium* associated bladder cancer: First report from Upper Egypt

**DOI:** 10.1371/journal.pntd.0011258

**Published:** 2023-04-17

**Authors:** Sara Abdelal Mohammed, Helal F. Hetta, Asmaa M. Zahran, Mohammed E. M. Tolba, Rasha A. H. Attia, Hosny M. Behnsawy, Abdelazeem M. Algammal, Gaber El-Saber Batiha, Ahmed Qasem Mohammed, Alzahraa Abdelraouf Ahmad

**Affiliations:** 1 Department of Parasitology, Faculty of veterinary medicine, Assiut University, Assiut, Egypt; 2 Department of Medical Microbiology and Immunology, Faculty of Medicine, Assiut University, Assiut, Egypt; 3 Department of Clinical Pathology, South Egypt Cancer Institute, Assiut University, Assiut Egypt; 4 Department of Parasitology, Faculty of Medicine, Assiut University, Assiut, Egypt; 5 Department of Urology, Faculty of Medicine, Assiut University, Assiut, Egypt; 6 Department of Bacteriology, Immunology, and Mycology, Faculty of Veterinary Medicine, Suez Canal University, Ismailia, Egypt; 7 Department of Pharmacology and Therapeutics, Faculty of Veterinary Medicines, Damanhour University, Damanhour, Egypt; 8 Department of Gastroenterology, Hepatology and infectious diseases, Al-Azhar University, Assuit, Egypt; INGEBI, ARGENTINA

## Abstract

**Background:**

The function of different populations of the immune system in bladder cancer (BCa) is well established. However, the cohesive role of the immune cell profile of schistosomal BCa at systemic and tissue levels is still lacking, especially in endemic countries. The balance hypothesized between protumorigenic and antitumor molecules determines the prognosis of tumor progression. This study aimed to investigate the frequency of T cell subsets at both blood and tumor tissue, regulatory T(Treg), regulatory B cells (Breg) and proinflammatory cytokines in *S*. *haematobium*-related BCa patients in Egypt.

**Methodology/Principal findings:**

The frequency of T cell subsets at both blood and tumor tissue, regulatory T(Treg), regulatory B cells (Breg) were studied by flow cytometry and proinflammatory cytokines by ELISA in *S*. *haematobium*-related BCa patients in Egypt. The results indicated a significant increase in the activity of T-cell populations, particularly CD3^+^, CD4^+^, and regulatory T cells (Tregs), and a decrease in cytotoxic CD8^+^ T cells in the patient group. An increased proportion of CD19^+^CD24^+^CD38^+^ Bregs and proinflammatory cytokines (IL-1β, IL-6, and TNF-α) was also observed. However, T-cell subpopulations in the tumor microenvironment showed a significant reduction in cancer patients compared to controls. Moreover, positive correlations were observed between the frequencies of Bregs and Tregs, suggesting the promotion of cancer progression besides their relation to the intensity of schistosomal infection.

**Conclusions/Significance:**

Trapped *Schistosoma haematobium* eggs in bladder tissue might lead to persistent inflammation that contributes to immunomodulation and promotes tumor progression, as evidenced by the increase in peripheral T helper, Tregs, Bregs and serum tumor-promoting cytokines. Considering the role and integrated functions of specific immune responses in BCa could help future diagnostic and therapeutic implications.

## Author summary

Bladder cancer was found to be associated with *S*. *haematobium* infection which is an endemic parasitic infection in many tropical and subtropical areas including Egypt. Toxic products released by *Schistosoma* eggs serve as antigenic triggers for the immune response. Several studies discussed the function of both resident and recruited immune cell response in BCa as the process of immunoediting in tumor progression is crucial in modulating the clinical course of tumors together with the characteristics of the tumor itself. This study is the first to report the immune cell profile at both epithelial and hematological levels in *S*. *haematobium*-related BCa patients in Upper Egypt to explore their immune status. This study would provide evidence of the changes in the proportions of T and B lymphocyte populations in cancer patients relative to the current infection state along with the proinflammatory cytokine profiles of IL-1, IL-6, and TNF-α. This study emphasized the role of T helper immune response in schistosomal BCa even under conditions of general immune suppression elicited by cancer and the inhibition of the cytotoxic immune response (CD8^+^) and generation of Tregs (CD4^+^CD25^high^Foxp3), resulting in the suppression of antitumor immunity. Additionally, significant Breg expression in patients seems to be closely linked to increased Treg cell production either peripherally or in the tumor microenvironment (TME), enhancing the conversion of nonactivated CD4^+^ cells into Tregs, with a decline in cytotoxic T-cell response in tumor tissue. At the TME, the past chronic inflammation elicited by schistosome egg antigens could be postulated to affect the suppression that occurred in T-cell subsets, thus promoting tumor progression. Also, the profound increase in the serum levels of proinflammatory cytokines correlates with the intensity of schistosomal infection in bladder tissue and suggesting a possible role in malignant transformation.

## Introduction

Urogenital schistosomiasis caused by *Schistosoma haematobium* infection represents a significant debilitating disease in many tropical and subtropical regions in sub-Saharan Africa, Sudan, Egypt, and Yemen, affecting more than 200 million people worldwide [1]. Unlike the widely studied *Schistosoma mansoni* and *Schistosoma japonicum*, with low evidence of their carcinogenic potential, *S*. *haematobium* infection is long known for its potential risk for bladder cancer (BCa; Group 1, human carcinogens) according to the World Health Organization’s (WHO) International Agency for Research on Cancer [2,3].

BCa is one of the most prevalent cancers, especially in men worldwide. Squamous cell carcinoma (SCC) is commonly present in rural Africa due to the endemicity of *S*. *haematobium* infection [4]. In contrast, most patients in developing countries and nonendemic regions presented with urothelial or transitional cell carcinoma (UC) [5]. Many retrospective studies have documented the relation between urogenital schistosomiasis and BCa in different endemic areas in the Middle East and Africa, including Egypt [6–8]. Schistosomal BCa is responsible for approximately 150,000 annual deaths, with an estimated treatment cost of 20 million USD worldwide, increasing the need for treating urogenital schistosomiasis and raising inquiries about the mechanism by which *S*. *haematobium* contributes to the development of BCa [9].

Most of pathology of urogenital schistosomiasis is caused by *Schistosoma* egg deposition in bladder tissue and eventually the host immune response against trapped eggs [10]. Consequently, inflammation, granulomas with further fibrosis, and bladder dysfunction will develop. In addition, the intervening pathogenesis can produce genotoxic factors that cause a proliferative response and oncogenic activity or inactivating tumor suppressor genes, increasing the susceptibility of malignant transformation in *Schistosoma*-infected patients [4,11,12]. A crucial player in *S*. *haematobium* pathogenesis is the cellular immune response chiefly regulated by T helper cells and stimulated by *Schistosoma* egg antigens [13].

The function of both resident and recruited immune cell response in BCa is a subject of interest of many researchers. The immunoediting process in tumor progression hypothesized the mechanism by which protumor and antitumor immune molecules can modulate the clinical course of tumors together with the characteristics of the tumor itself [14]. It also highlights the function of immune regulation concerning regulatory T cells (Tregs) that exhibit an immunologic self-tolerance, inactivating other T cells, and promote tumor progression [15–17]. Tregs express different cell surface markers that differentiate them from activated T cells, such as CD25^high^ and Foxp3, described as a chief transcription factor specific for Tregs [18].

Furthermore, B cells support the development of a strong Th2 response associated with helminth infections [19] and are involved in the regulatory functions of T cells in schistosomal infections [20]. Also, they augment T-cell conversion into Tregs with a subsequent decline in antitumor activity [21]. Further, researchers have postulated different mechanisms of regulatory B cells (Bregs) in promoting cancer progression and metastasis. B cells generate antibodies and immune complex deposition, fueling chronic inflammation and inducing angiogenesis in preneoplastic and neoplastic tumor tissue [22,23].

Remarkably, the cytokine profile and immune cell activation milieu during the antitumor immunological phase in tumor development could define the outcomes of the host-tumor interface. Interleukin (IL)-1β and IL-6 are proinflammatory cytokines involved in cancer progression. In BCa patients, decreased IL-1α mRNA expression was correlated with a low patient survival rate [24]. IL- 6 was described as one of the tumor-promoting factors that induce the epigenetic transition from non-transformed epithelia into neoplastic cells, affecting tumor development and metastasis [25–27]. Also, the increased risk of BCa with or without schistosomal infection was linked to the expression of the inflammatory mediator tumor necrosis factor-α (TNF-α) [28,29]. TNF-α also promoted tumor invasion and metastasis by stimulating matrix metalloproteinase-9 secretion [30]. Therefore, continuing studies could enhance the understanding of cancer bladder development by investigating immune regulatory cells, transcription factors, and polymorphic genes.

This study aimed to investigate the immune cell profile at both blood and tumor tissues in *S*. *haematobium*-related BCa patients in Egypt to explore their immune status. This study would provide evidence of the changes in the frequency of lymphocyte populations in cancer patients relative to the current infection state for future diagnostic and therapeutic approaches. Also, IL-1, IL-6, and TNF-α were evaluated as they are considered key mediator cytokines of the inflammatory milieu and modulate tumor-promoting factors.

## Methods

### Ethics statement

The study was approved by our ethical committee of the Faculty of Medicine, Assiut University (IRB no. 17300818) according to the declaration of Helsinki II (1975). Informed written consent was obtained from all participants in the study.

### Study subjects

A case-control study was performed at the Parasitology Department and Urology Hospital of Assiut University (Egypt) from July 2017 to June 2018. Fifteen BCa patients admitted to the hospital for cancer resection or biopsy were enrolled in the study. Only patients with a history of chronic *S*. *haematobium* infection were included in the study. Patient criteria included a history of hematuria, radiological findings suggesting malignancy by either ultrasound or computed tomography scan, and positive urine cytology. Patients with metastatic tumors were excluded from the study. All participants were treated for urinary tract infections before the study.

**Fig 1:**
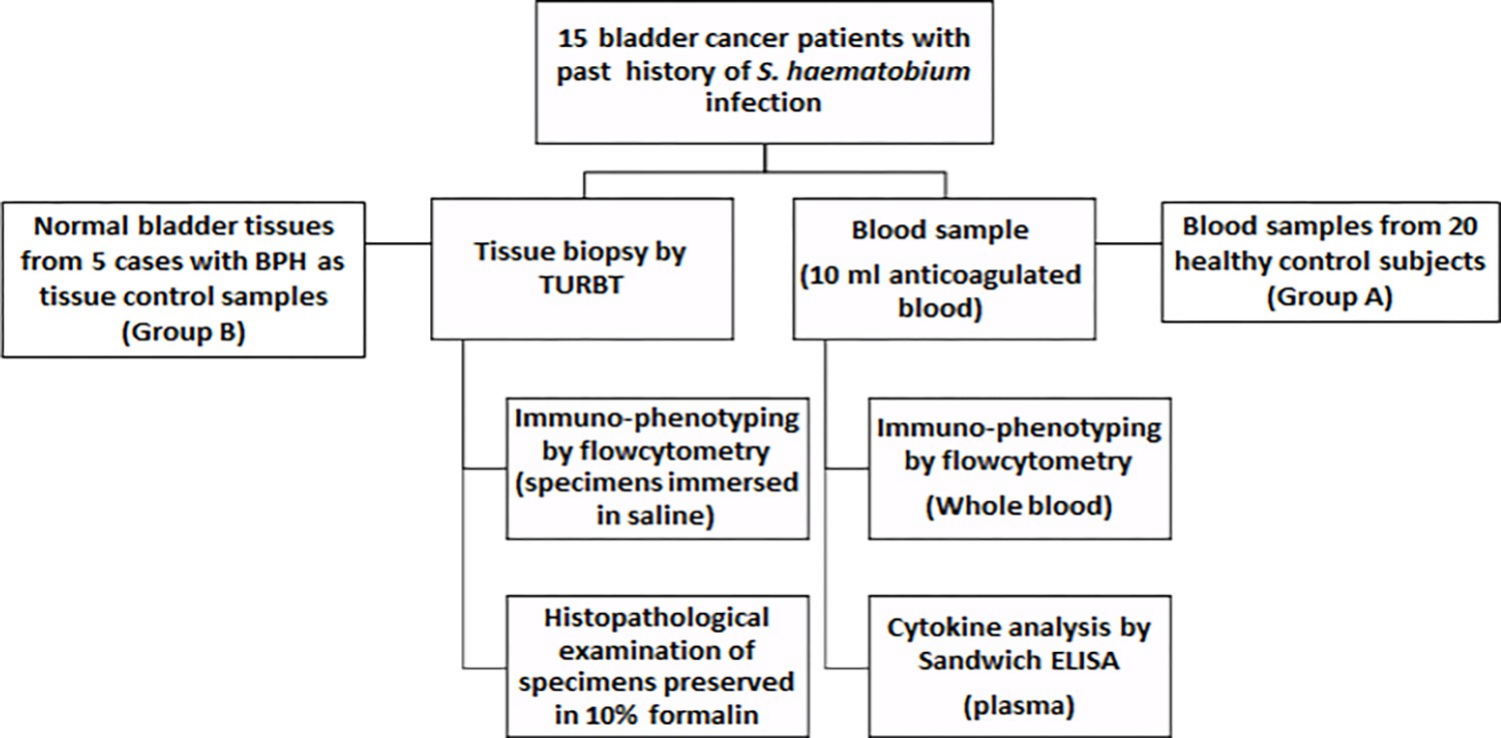
Study flowchart.

Two control groups were included in the study. Group A included 20 noninfected subjects (19 males and 1 female) ages 25 to 55 years; they were healthy laboratory personnel who volunteered to participate in this study and donate blood sample for hematological study. A second control group (group B) was used for assessment of cancer bladder tissues. Normal bladder tissues were collected from five patients who have been referred to urology hospital to perform TURB for benign prostatic hyperplasia (BPH). All control subjects were tested for endemic parasitic infections, including *S*. *haematobium*. Diabetic subjects were also excluded **(Fig 1**).

### Sample collection and processing

#### Blood samples

Anticoagulated blood samples (10 ml) on EDTA sterile tubes were collected from both patients and the control group A. They were divided into two sterile tubes: one for flow cytometric analysis and the other for cytokine measurement by sandwich enzyme-linked immunosorbent assay (ELISA). All samples were transported immediately to the laboratory at room temperature.

#### Tissue biopsy

Bladder tissue biopsies were collected from BCa patients by either transurethral resection of bladder tumor (TURB) or partial cystectomy. Normal bladder tissue samples collected from the control group B (BPH) were involved in this study and used as control tissue. Biopsy specimens were divided into two parts, 2 to 3 g each, in separate sterile containers. The first part was preserved in 10% formalin for histopathological examination, and the second part was immersed in saline solution for flow cytometric analysis.

### Histopathological examination

Bladder tissue specimens were processed for histopathological examination to determine the grade, stage and other histopathological characteristics. Grading of BCa was done according to the World Health Organization/International Society of Urologic Pathology classification of urothelial neoplasia [31]. The pathological staging and invasiveness of cancer were assessed based on American Joint Committee on Cancer Classification [32]. Briefly, small parts of the tissue were fixed in 10% formalin, dehydrated, and embedded in paraffin. Tissue sections (5 μm) were dewaxed, stained with hematoxylin & eosin (H&E), and permanently mounted in Canada balsam. Using a light microscope under different magnifications, the stage and grade of cancer were recorded.

### Flow cytometric detection of T- lymphocytes and regulatory T cells

T-lymphocytes were detected using 100 μl of blood sample, which was stained with 10 μl of fluoroisothiocyanate (FITC)-conjugated CD3, Peridinin Chlorophyll Protein Complex (PerCP)-conjugated CD4 and phycoerythrin (PE) conjugated CD8 antibodies (Becton Dickinson Biosciences, USA).

**Fig 2:**
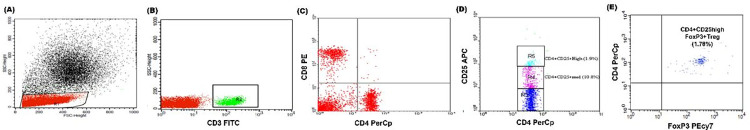
Flow cytometric detection of T- lymphocytes and regulatory T cells. A: Forward and side scatter plot was used to define the lymphocyte population (R1). B and C: The expression of and CD3 (R1), CD4^+^, and CD8^+^ was assessed in lymphocytes population. D: CD4^+^ T cells were gated and the expression of CD25 in CD4^+^ T cells was detected, compared with the negative isotype control (not shown) and different gates were used to define CD4^+^ CD25^-^ cells (R3), CD4^+^CD25^+intermediate^ (med) cells (R4), and CD4^+^CD25^+High^ cells (R5). E: The percentage of CD4+CD25^+high^ FoxP3+cells in CD4^+^ T cells was determined and considered as Treg cells.

Tregs were enumerated using PerCP-conjugated CD4 (Becton Dickinson, Bioscience, USA), APC-conjugated CD25 (IQ Product, The Netherland) and Foxp3-PEcy7 (e-Biosχienχe, USA). Flow cytometric analysis was performed using the Fluorescence Activated Cell Sorter (FACS) Canto II Flow Cytometry system with Cell Quest software (Becton Dickinson Biosciences, USA). Isotype control of anti-human IgG was used as a negative control for each sample. Forward and side scatter histogram was used to define the lymphocyte population and the percentages of CD3^+^ (T-lymphocytes), CD4^+^ (T-helper cells) and CD8^+^ (T-cytotoxic) were assessed from the lymphocyte population. In addition, CD4^+^CD25^-^, CD4^+^CD25^+intermediate^, CD4+CD25^+high^, and CD4^+^CD25^+high^ Foxp3^+^regulatory T cells were evaluated on both CD4^+^. The mean fluorescence intensity (MFI) was used for Foxp3 expression as shown in **Fig 2**.

### Flow cytometric analysis of T lymphocytes, T helper, and T cytotoxic cells in Bladder cancer tissue

Biopsy tissue samples were obtained for immunophenotyping of T-cell surface markers by flow cytometry according to Lanier et al. (1986) and McMichael et al. (1987). Briefly, tumor biopsies were cut into small fragments of 1 to 2 mm, ground, and mixed with PBS. They were strained through a 200 μm mesh strainer at room temperature, centrifuged, washed with PBS + 0.1% BSA, and then resuspended (PBS + 0.1% BSA). The cells were adjusted to about 10,000 cells/ml PBS [33,34]. Phenotypic antigen expression on tissue T lymphocytes was performed using the following antibodies: FITC-conjugated CD3, PerCP-conjugated CD4 and PE conjugated CD8 antibodies. The samples were processed and analyzed as described previously in blood samples.

### Evaluation of the frequency of peripheral B lymphocytes and Bregs by flow cytometry

**Fig 3:**
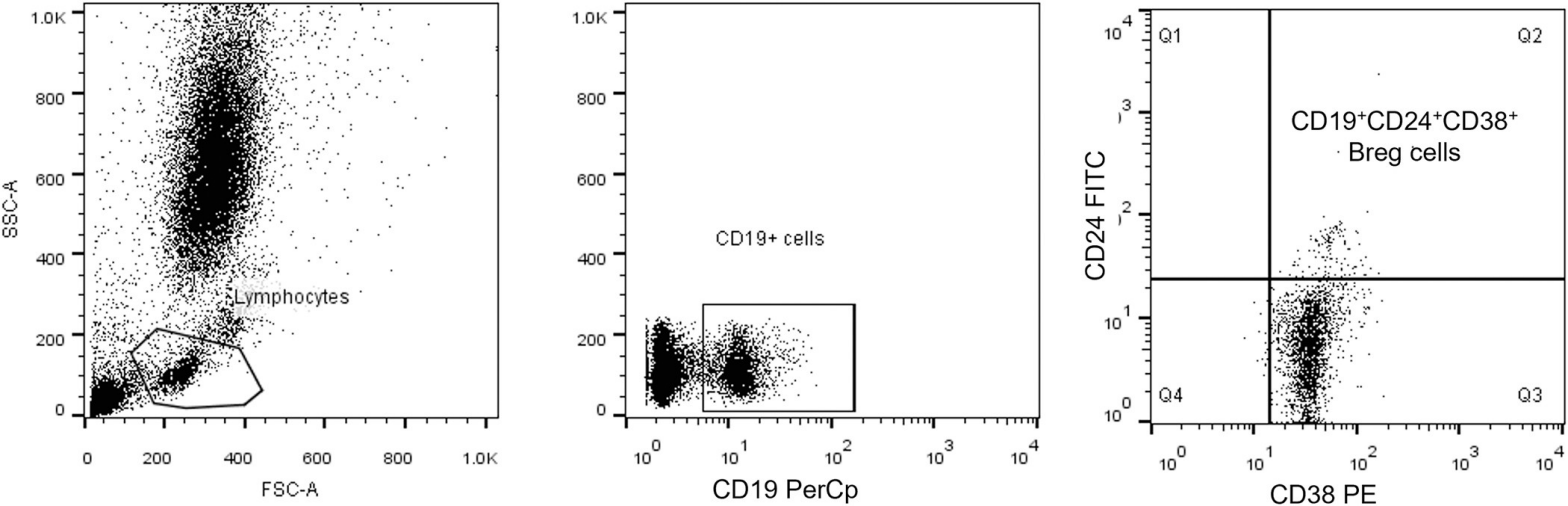
Flow cytometric detection of regulatory B cells. **A**: Scatter histogram was used to define the lymphocytes population. **B**: The CD19^+^ cells were assessed within the lymphocyte population and then gated for further assessment of CD24 and CD38. **C**: The expression of CD24 and CD38 was assessed in CD19^+^ lymphocytes to define CD19^+^CD24^+high^CD38^+high^ cells (regulatory B cells).

Circulating B lymphocytes and Bregs were detected in peripheral blood using the following markers (BD Biosciences, San Jose, CA, USA): PerCP-conjugated CD19, FITC-conjugated CD24 and PE-conjugated CD38. Briefly, the blood sample (100 μl) was incubated with 10 μL of the markers (CD19,CD24 and CD38) for 20 min at 4°C in the dark. RBCs were lysed using BD FACS Lysing solution (BD Biosciences, San Jose, CA, USA), and washing was done after incubation. Gating of CD19^+^ B cells was done, followed by CD38^+^ and CD24^+^ expression on CD19^+^ B cells. The analysis was done as described previously. CD19^+^CD24^+hi^CD38^+hi^ cells were identified as Bregs. (**Fig 3)**

### Measurement of serum levels of proinflammatory cytokines (IL-1β, IL-6, and TNF-α)

Sandwich ELISA test was done to measure the serum levels of proinflammatory cytokines (IL-1β, IL-6, and TNF-α) using ELISA kits purchased from BD Biosciences (for IL-1β and IL-6) and Biolegend (for TNF-α) according to the manufacturer’s recommendations. The tests were performed in duplicate. The mean absorbance of standards, controls, and samples was calculated. The lower detection limit was determined to be 2.5 to 5.0 pg/ml according to the estimated cytokine.

### Statistical analysis

Data were verified and analyzed using IBM-SPSS software version 20.0 (SPSS, Chicago, IL, USA). All quantitative data were presented as median and range. Mann-Whitney U test, Kruskal-Wallis test, and Wilcoxon matched-pairs test were calculated for nonparametric data analysis. Pearson’s correlation test and Fisher’s exact tests were used for categorical data. GraphPad Prism was also used. *p* < 0.05 was considered significant.

## Results

A group of 15 patients admitted to the Urology Hospital of Assiut University presented with clinical features suggestive of BCa. Those patients gave a history of previous *S*. *haematobium* infection and received praziquantel treatment in the past. The demographic criteria and histopathological findings of the patients are summarized in Table 1. Most patients were males [13 (86.7%)], ages 47 to 73 years (mean, 55.73 ± 7.5 years).

**Table 1:** Demographic criteria and histopathological findings of the studied patients.

Patient No.	Demographic criteria (Age/Sex)	Type of neoplasm	Cancer Grading	TNM staging*	Intensity of *Schistosoma* egg deposition	Type of biopsy
1	66 year old /Male	^a^ UC	High grade	pT2a	Few dead eggs	TURBT
2	60/ Male	UC	High-grade ^b^ invasive with areas of squamous differentiation.	pT2a	Heavy dead eggs	TURBT
3	57/ Male	UC	High-grade	pT2a	Few calcified eggs	TURBT
4	50/ Male	UC	Low-grade	pT1a	Few dead eggs	TURBT
5	51/Male	UC	High-grade	pT2a	Heavy dead eggs	TURBT
6	59/ Male	UC	High-grade	pT2a	Heavy dead eggs	TURBT
7	50/ Male	UC	High-grade	pT2a	Few dead eggs	TURBT
8	52/ Female		High-grade ^b^ with areas of squamous differentiation	pT2a	Few dead eggs	TURBT
9	52/ Male	UC	High-grade ^b^ with areas of squamous differentiation	pT2a	Few dead eggs	TURBT
10	49/ Female	UC	High-grade and invasive ^b^ with areas of squamous differentiation	pT2a	Few dead eggs	TURBT
11	47/ Male	Signet ring cell carcinoma	High-grade	pT2a	Few calcified eggs	TURBT
12	54/ Male	SCC	High-grade	pT2a	Heavy dead eggs	TURBT
13	73/ Male	SCC	Invasive high-grade	pT3a	Few calcified eggs	TURBT
14	65/ Male	UC	High grade and invasive	pT2a	Heavy dead eggs	TURBT
15	51/ Male	UC	Invasive high-grade	pT2a	Few dead eggs	Partial cystectomy

*TNM staging according to AJCC (2002)[32]

^a^ UC: Urothelial carcinoma, SCC: Squamous cell carcinoma.

^b^ mixed UC with Squamous cell differentiation.

### Histopathological examination of cystoscopic biopsies

The histopathological examination of all specimens revealed multiple tissue fragments of neoplasm along with bilharzial egg granulomas detected in all samples. The results indicated the presence of urothelial carcinoma (UC) in most samples [12 (80%)]. Four cases (26.7%) of UC were mixed with areas of squamous differentiation (SCC), and the tumor sheets were infested with desmoplastic stroma and dead bilharzial eggs. The remaining three cases include two cases with SCC and one case with signet ring carcinoma which is a rare type of adenocarcinoma with pools of extracellular mucin and calcified bilharzial ova.

**Fig 4:**
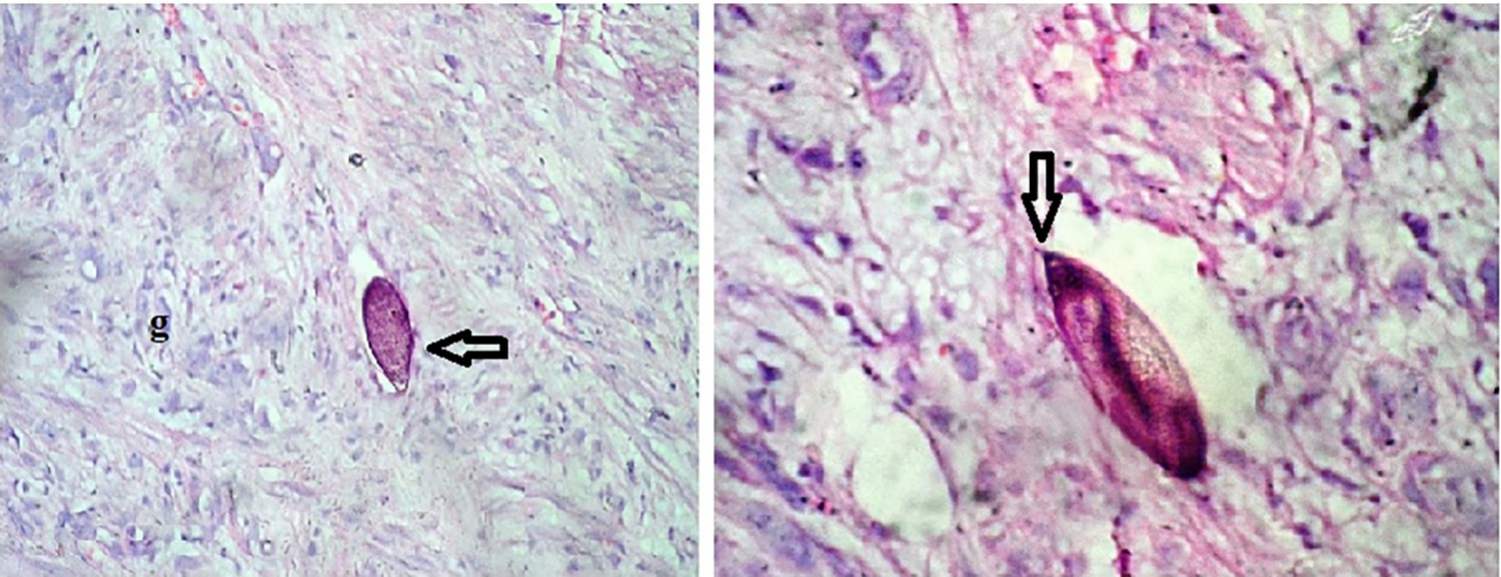
UC showing loose granulomas (g) around terminally spined *S*. *haematobium* eggs (black arrow) stained with H&E (×200).

The histopathological examination of bladder biopsies showed 14 of 15 cases of high-grade invasive neoplasm infiltrating the muscular layer, whereas only one case of low-grade neoplasm. Loose granulomas with dispersed lymphocytes and scarce fibrous tissue were detected. Also, variable patchy granulomas around multiple calcified and dead *Schistosoma* eggs were observed (Fig 4). The intensity of *Schistosoma* egg infection differed among cases (Table 2).

**Table 2:** Pathological data of the studied cases.

Variable		Total No. = 15
Type of neoplasm	UC	8 (53.3%)
	Mixed UC and SCC	4 (26.7%)
	SCC	2 (13.3%)
	Signet Ring	1 (6.7%)
Detected eggs	Few dead	7 (46.1%)
	Heavy dead	5 (33.3%)
	Few calcified	3 (20%)

### Lymphocyte subsets and Treg cells in *S*. *haematobium-*infected BCa patients

The characteristics of lymphocyte populations in *S*. *haematobium*-infected BCa patients were explored. The proportions of total lymphocytes, T and B-cell subsets in PBMCs, and tumor tissues were analyzed via multicolor flow cytometry. The total lymphocyte count was significantly lower in patients than controls (40.5% (20–53) vs. 55% (27–71); *p* = = 0.015). Also, there was a slight nonsignificant decline in CD8^+^ (cytotoxic T) cells in patients (*p* = 0.049). Meanwhile, BCa patients exhibited a significant increase in the total percentage of circulating T lymphocytes (CD3^+^) and T helper cells (CD4^+^) (70% (56–78) vs. 57% (39–74) and 39% (32–83) vs. 35% (28–62), respectively), with a statistically significant difference between the compared groups (*p* < 0.001 and *p* < 0.038, respectively; **Table 3).**

**Table 3:** Frequency of lymphocytes subsets and Treg cells among patients and healthy controls.

Median (Range)	Cases (n = 15)	Control (n = 20)	*P*-value
Total lymphocyte	40.5 (20–53)	55 (27–71)	= 0.015*
% CD3^+^ T cells	70 (56–78)	57 (39–74)	< 0.001*
% CD4^+^ T cells	39 (32–83)	35 (28–62)	= 0.038*
% CD8^+^ T cells	10.5 (3–29)	15 (5–36)	= 0.049
% CD4+CD25^High^Foxp3+ Treg	1.8 (1–3.5)	1.3 (0.5–1.5)	< 0.001*

= Mann-Whitney U test was used to compare the median between cases and controls.

Concerning the frequency of Tregs in both groups, a significant increase in *S*. *haematobium*-infected BCa patients was detected compared to controls (*p* < 0.001; **Table 3 and S1 Fig**).

### Correlation between the severity of schistosomal infection and T-cell subpopulations

The correlation between the intensity of schistosome egg deposition reflecting the severity of the infection and different T-cell subsets was evaluated. Schistosome-infected BCa patients were divided into mildly and heavily infected groups. The median of the total lymphocytes, CD3^+^, CD4^+^, CD8^+^, and Tregs was calculated in both groups. There was a nonsignificant correlation between the intensity of egg deposition and the level of a peripheral T-cell subpopulation (**Table 4**).

**Table 4:** Relationship between severity of infection and frequency of T lymphocyte subsets and Treg cells.

Median (Range)	Infection		*P*-value*
	Mild/Moderate	Heavy	
% CD3^+^ T cell	68 (56–78)	75.5 (65–78)	= 0.102
%CD4^+^ T cell	37.5 (34–77)	50 (32–83)	= 0.142
%CD8^+^ T cell	11 (3–24.5)	12 (7–29)	= 0.948
%Treg	1.8 (1.2–2)	1.9 (1.1–3.5)	= 0.864

*Mann-Whitney U test was used to compare the median between cases and controls

### Analysis of T-lymphocyte subsets in the tumor microenvironment by flow cytometry

BCa tissue samples were used to study the activity of T-lymphocyte subsets in the tumor microenvironment (TME), comparing the schistosome-infected (BCa) group and normal bladder tissues from BPH patients as control. In this study, the frequency of T-lymphocytes and their subsets in the schistosomal BCa group showed reduction in the level of total lymphocytes (CD3^+^), T helper (CD4^+^) cells, and cytotoxic T (CD8^+^) cells in tumor tissues compared to controls (group B) that was statistically significant (*p* < 0.001) (**Table 5)**.

**Table 5:** The differences in tissue T-cell subsets between patients and controls.

Median (Range)	Cancer Patients (n = 15)	Control group B (n = 5)	P-value*
%Tissue CD3^+^ T cells	10 (8–13)	20 (19–22)	< 0.001
%Tissue CD4^+^ T cells	8.5 (8–9.5)	16.5 (14–17.5)	< 0.001
%Tissue CD8^+^ T cells	1.4 (1–1.8)	2 (1.8–2.3)	< 0.001

*Mann-Whitney U test was used to compare the median between cases and controls

### Analysis of peripheral B cells and Bregs in BCa patients

To investigate schistosome-induced alterations in B-cell subpopulations in BCa patients, circulating B-cell subsets were analyzed among patients compared to healthy controls (group A). The proportion of total peripheral B lymphocytes showed no statistically significant difference between schistosome-infected cancer patients and healthy donors (13.23% vs. 14.22%, respectively; *p* = 0.316). On gating of CD19^+^ B cells, the expression of markers, CD38^+^ and CD24^+^ was detected, and Bregs were recognized as CD19^+^CD24^+hi^CD38^+hi^. Bregs were higher in patients than controls (5.84% vs. 3.55%; *p* = 0.001) (S2 Fig).

### Correlation of the frequency of Bregs with T-cell subsets among BCa patients

As shown in Table 6, the frequency of peripheral Bregs was positively correlated with the frequency of peripheral CD3^+^ (*r* = 0.297; *p* = 0.041), CD4^+^ (*r* = 0.400; *p* = 0.009), and Tregs (*r* = 0.258; *p* = 0.048); however, circulatory CD8^+^ revealed no significant correlation with Bregs (*r* = 0.067; *p* = 0.352). At the TME level, Bregs were only positively correlated with tissue Tregs (*r* = 0.502; *p* = 0.021) and tissue cytotoxic CD8^+^ cells (*r* = 0.391; *p* = 0.041).

**Table 6:** Correlation between Bregs level and peripheral T-cell subpopulations.

	Breg cells	*P*-value**
	rho*	
Blood CD3 T cells Tissue CD3 T cells	0.297 0.171	0.041** 0.271
Blood CD4 T cells Tissue CD4 T cells	0.400 0.082	0.009** 0.386
Blood CD8 T cells Tissue CD8 T cells	0.067 0.391	0.352 0.041**
Blood Treg cells Tissue Treg cells	0.258 0.502	0.048** 0.021**

*Spearman Rank correlation coefficient

**Based on normal approximation.

### Plasma cytokine analysis in relation to infection

To explore the function of circulatory proinflammatory cytokines and identify its relationship with the patient’s current infection status and the role of *S*. *haematobium* chronic inflammation in BCa, the plasma cytokine levels of IL-1β, IL-6, and TNF-α were measured in both patients and control group A. The cytokine titers for IL-1β, IL-6, and TNF-α were higher, up to fourfold, in patients than in controls (**Table 7**). Further analyses of the cytokine profile and its association with other immune cells were done. Spearman’s correlation analysis between cytokines and T-cell surface markers indicated no significant correlation between the measured cytokines and the T-cell subsets of CD3^+,^ CD4^+^, and Tregs; however, IL-1β showed a significant positive correlation with CD8^+^ (*r* = 0.408; *p* = 0.026), as shown in **Table 8**.

**Table 7:** Differences in the proinflammatory cytokine levels between patients and control group A.

Median (Range)	Patients (n = 15)	Control group A (n = 20)	*P*-value**
IL1 (pg/ml)	26 (23–27.5)	5 (4–5.5)	< 0.001
IL6 (pg/ml)	37 (35–38.5)	8.5 (8–11)	< 0.001
TNF-α (pg/ml)	220 (215–225)	50 (48–55)	< 0.001

*Mann-Whitney U test was used to compare the median between cases and controls

**Table 8:** Correlation among immune cells, cytokine levels, and the severity of infection among patients.

	IL1		IL6		TNF-α	
	Rho*	*P*-value**	rho	*P*-value**	rho	*P*-value**
Blood CD3^+^ T cells	0.268	= 0.167	0.329	= 0.115	0.192	= 0.247
Blood CD4^+^ T cells	0.039	= 0.444	-0.125	= 0.328	-0.095	= 0.368
Blood CD8^+^ T cells	0.408	= 0.026**	-0.109	= 0.349	0.225	= 0.210
Blood CD25^+^ T cells	-0.050	= 0.429	-0.303	= 0.136	-0.011	= 0.485
Severity of infection	0.539	= 0.029**	0.495	= 0.043**	0.660	= 0.007**

*Spearman Rank correlation coefficient

**Based on normal approximation.

The relationship between the intensity of *S*. *haematobium* infection in BCa patients and their serum levels of proinflammatory cytokines was assessed. The infection intensity based on schistosome egg deposition versus cytokine levels indicated that all measured cytokines had a significant relationship with the intensity of infection. TNF-α, IL-1β, and IL-6 levels showed a significant positive correlation with the severity of infection (*r* = 0.660, 0.539, and 0.495, respectively; **Table 8**). Hence, high egg counts were linked with high cytokine levels.

## Discussion

The relationship between urogenital schistosomiasis and increased risk of BCa is well established in which bladder tissue showed different changes at gross morphological, molecular, and immunological levels [35]. Several clinical trials were conducted to determine the associations and confirm clinical evidence that helps explore the mechanism of schistosomal BCa. Therefore, this study aimed to elucidate the relevance of the immune profile at both epithelial and hematological levels in schistosome-infected BCa patients in Upper Egypt.

This study highlighted the predominance of BCa in male patients that concur with the WHO and SCR data that describe BCa as a disease of the male gender [36]. Recent studies confirmed this observation, including that conducted in Egypt [37].

The results revealed the presence of UC with or without squamous differentiation in mostofthe studied cases besides SCC and signet cell ring carcinoma with many scattered calcified and/ or dead *S*. *haematobium* eggs. These findings were similar to previous studies that reported *S*. *haematobium* eggs in more than 85% of BCa tissue samples [38,39]. An Egyptian study documented that BCa accounted for 30.3% of all reported cancers, and most of them were SCC attributed to *S*. *haematobium* infection [40]. This was matched to other African studies from Sudan, Kenya, Uganda, Nigeria, and Senegal [41]. However, due to the small sample size in the present study, we could not conclude the predominance of one type of neoplasms over the other which requires multicenter studies on more patients to detect the relationship between the type of cancer and *S*. *haematobium* infection in Upper Egypt.

Although *S*. *haematobium* infection is well known as a major risk factor of BCa, especially in endemic countries, the integrated functions of different immune cell populations in schistosomal BCa at systemic and tissue levels are still understudied. To investigate the immunologic status triggered by different infectious diseases, immunophenotypic analysis of lymphocytic populations by flow cytometry was determined as conclusive [42]. The phenotype profiles of T and B lymphocytes of BCa patients with chronic *S*. *haematobium* infection and healthy subjects were investigated. Total lymphocytes were significantly decreased, reflecting the general immune suppression in cancer patients and the chronicity of schistosomiasis. These data agreed with previous reports that showed a reduction in total lymphocytes in the chronic phase of *S*. *mansoni*-infected patients [43]. Also, these results reflected a state of systemic immunosuppression in BCa patients, as described by earlier studies [44,45].

This study described a significant increase in the frequency of both CD3^+^ and CD4^+^ cells in peripheral blood of chronic *S*. *haematobium*-infected patients who developed bladder cancer, demonstrating a possible correlation. These results indicated the activity of T-cell subpopulations in the present immune milieu. The elevated levels of CD3^+^ and CD4^+^ cells could be attributed to the continuous stimulation of cellular immune response produced by trapped schistosome egg antigens, maintaining the inflammation around the eggs. These observations agreed with Martins-Filho et al. 1997, who stated that the general immune suppression in chronic late hepatocellular fibrosis from old *S*. *mansoni* infection was not accompanied by a decrease in circulating CD3^+^ cells [43]. Moreover, previous studies reported a significant increase in T-cell populations, which correlated with disease chronicity in older schistosomal infections [46,47].

Despite the significant increase in CD3^+^ and CD4^+^ cells in this study, cytotoxic CD8^+^ cells were lower in patients than controls. This also agreed with previous similar reports that showed an increase in activated CD4^+^ cells with no significant alterations in the value of CD8^+^ cells [43,48]. Furthermore, Agarwal et al. [45] documented that CD8^+^ and natural killer (NK) cells showed a significant decline in BCa patients with subsequent suppression in cytotoxic immune response and improper lysis of tumor cells in patients, attenuating antitumor immunity.

In this study, the percentages of peripheral T-cell subpopulations showed no relationship with the intensity of bladder infection with *Schistosoma* eggs, as reported previously [47]. Remarkably, Tregs are crucial for maintaining peripheral tolerance in chronic inflammatory diseases [49]. Also, they have beneficial properties in the TME. Tregs induce a potent immunosuppressive function by inhibiting the antitumor immune response and augmenting tumor growth. Tregs are involved in tumor evasive strategies through different mechanisms, promoting cancer cell survival [50–52].

Herein, our results showed that BCa patients exhibited a significant increase in Tregs in peripheral blood, which are consistent with previous reports on different types of cancer that showed increased Tregs peripherally or in tumor tissue [53–55]. The correlation of Treg proportion with the BCa stage could not be assessed because of the limited sample size

In the same context, the infiltration of T-cell subpopulation in tumor tissue showed a significant decrease in tissue CD3^+^, CD4^+^, and CD8^+^ cells. These cells were recognized as important players in antitumor immunity [56]. The infiltration of these cells to TME was reported in previous studies and mostly indicated a good prognosis [57]. Based on histopathological examination, most cases in the present study were high-grade invasive tumors and this could be postulated the main cause of the depletion of T lymphocytes and their subpopulations.

As documented previously, many researchers have elucidated the role of Tregs in cancer progression, yet the function of Bregs in modulating the immune response of BCa is controversial. In this study, CD24^hi^CD38^hi^CD19^+^ Bregs were evaluated in schistosome-infected BCa patients compared to healthy controls and demonstrated a significant increase in Bregs in patients. The proportion of Bregs was positively correlated with peripheral T-cell subsets, including CD3^+^, CD4^+^, and Tregs, unlike circulatory CD8^+^. However, in the TME, Bregs positively correlated only with Tregs and cytotoxic CD8^+^ infiltrating cells.

Several murine models have investigated the potential mechanisms of B-cell functions in cancer immunity. Breg subsets revealed a significant regulatory function with primary inhibitory effects such as inhibiting cytotoxic T and NK cells [58]. B-cell depletion is currently documented with decreased tumor growth in multiple murine tumor models, impeding the inhibition of cytotoxic T-cell-mediated immunity or increased Treg production [59,60]. This is consistent with this study in which Bregs correlated with increased production of Tregs peripherally and in the TME, promoting the conversion of nonactivated CD4^+^ cells into Tregs. In addition, Bregs paradoxically induce a decline in cytotoxic T-cell response via low CD8^+^ expression in tumor tissue, with high-grade invasive cancer. However, other studies disagree with the findings of this study. Some reports showed that intratumoral B cells might be crucial in tumor cell destruction by enhancing T-cell responses or via antibody-dependent cellular toxicity [61–63].

The cytokine expression profiles during antitumor immunity could also determine the outcomes of host-tumor interaction. In *S*. *haematobium* infection, generated cytokines by Th1 and Th2 cells have a vital role in regulating cytotoxic T lymphocytes which are important immune cells in tumor regression. This study investigated the role of proinflammatory cytokines (IL- 1β, IL-6, and TNF-α) as key mediators in the promotion of carcinogenesis in schistosomal BCa. Herein, a significant increase in IL-1β, IL-6, and TNF-α serum levels was detected that could support the role of proinflammatory cytokines in cancer patients.

Interestingly, IL-6 was previously described in the regulation of cell proliferation, survival, and apoptosis, and IL-6 signaling was associated with tumorigenesis in several tumors, including BCa [25,64–66]. An increased level of IL-6 production was identified as a tumor-promoting factor that affects inflammatory milieu and Th2 immune cell response [67].

IL-1β has been demonstrated in chronic inflammation and is predominantly expressed in the TME by tumor-infiltrating macrophages, promoting tumor development and metastasis [68]. These data is consistent with our study, a positive correlation between IL-1β and cytotoxic CD8^+^ levels was detected. Also, a strong correlation between this cytokine and the intensity of schistosomal infection in bladder tissue was observed. Furthermore, elevated IL-1β levels described in this study agreed with several previous studies that stated that IL-1 acts as proangiogenic and prometastatic mediators in the TME in different tumors [69,70]. Hence, it could be postulated that chronic inflammatory condition provoked by *S*. *haematobium* infection triggers the ugly face of IL-1 with antiapoptotic and protumorigenic functions, promoting tumor development by different mechanisms [71,72].

Concerning TNF-α, the results revealed a fourfold increase in TNF-α serum levels that exceeded the difference observed in CD4^+^ cells between study groups. This is typically consistent with a previous study that showed that patients with bladder wall pathology exhibited a profound TNF-α production in PBMC cultures compared to CD4^+^ cell-enriched cultures. [73]. Also, this study implied a significant relationship between TNF-α serum levels and the severity of infection in heavily infected bladder tissue, indicating a strong association between antigens secreted by the trapped eggs and TNF-α serum levels. Remarkably, higher TNF-α levels were observed in advanced stages of BCa, indicating that TNF-α is implicated in BCa progression, promoting the invasion and migration of neoplastic cells [74,75]. Interestingly, all data highlighted a significant positive correlation between the severity of egg infection and increased IL-1, IL-6, and TNF-α levels, indicating the possible influence of *S*. *haematobium* egg antigen on tumor-promoting cytokine levels and supporting BCa development and progression.

### Study limitations and future recommendations

The sample size was the major limitation of this study. In addition, the methods for characterizing the inflammatory state of the patients were simple. It may have been helpful to perform PBMC or whole blood cell cultures or to measure additional cytokines in the serum and determine if proportions of each correlated with cancer grade of schistosome egg count. It is very important to understand the role of inflammation in the development of cancer. However, a critical control group is missing (schisto-infected only). The circulating cells could have been functionally characterized to increase our insight into their possible roles. only few cytokines were measured in the serum so more future work is recommended to do functional characterization of cells and performing comprehensive cytokine profile.

## Conclusion

This study suggested the possible role of T helper immune response in schistosomal BCa even under conditions of general immune suppression elicited by cancer and the inhibition of the cytotoxic immune response (CD8^+^) and generation of Tregs (CD4^+^CD25^high^Foxp3), resulting in the suppression of antitumor immunity. Additionally, significant Breg expression in patients seems to be closely linked to increased Treg cell production either peripherally or in the TME, postulating their role in the conversion of nonactivated CD4^+^ cells into Tregs, with a decline in cytotoxic T-cell response in tumor tissue. At the TME, the presence of trapped dead schistosome eggs with signs of previous chronic inflammation may be related to suppression in T-cell subsets, promoting tumor progression. Also, the profound increase in the serum levels of proinflammatory cytokines that correlates with the intensity of schistosomal infection in bladder tissue is possibly implicated inmalignant transformation. However, the relatively small sample size limited the scope of the study and the definite association between the grade and stage of schistosomal BCa and the immune cell profiles in those patients. Further studies are needed to identify the key components of schistosome-associated immunomodulation integrated with tumor-evasive strategies in bladder carcinogenesis for future diagnostic and therapeutic implications.

## Supporting information

S1 Fig Total lymphocytes and T cell subset analysis. The dot graph shows a significant reduction in the percentage of total lymphocytic count in BC patients while CD4^+^ showed a significant increase, however there is a mild reduction in CD8^+^ in patient group. The percentage of Treg cells shows a significant increase in the patient group than the control (P = 0.015, P = 0.038, P = 0.049, and P < 0.001, respectively) (Grey line represents the median).
(TIF)

S2 Fig B cell subsets analysis. The graph shows the percentages of total B lymphocytes and Breg cells in BCa patients and control. Medians are shown as gray lines and data are compared by a non-parametric Mann-Whitney-U test. The dot graph shows a significant increase in B regulatory cells in the patient group while the total B cell number is lower in patients versus the healthy control with no statistical significance.
(TIF)
