# Supplementary figures and images for: T cell subsets, regulatory T, regulatory B cells and proinflammatory cytokine profile in Schistosoma haematobium associated bladder cancer: First report from Upper Egypt
Source: PLoS Negl Trop Dis. 2023 Apr 17;17(4):e0011258. doi: 10.1371/journal.pntd.0011258 (PMC10109487; doi:10.1371/journal.pntd.0011258)

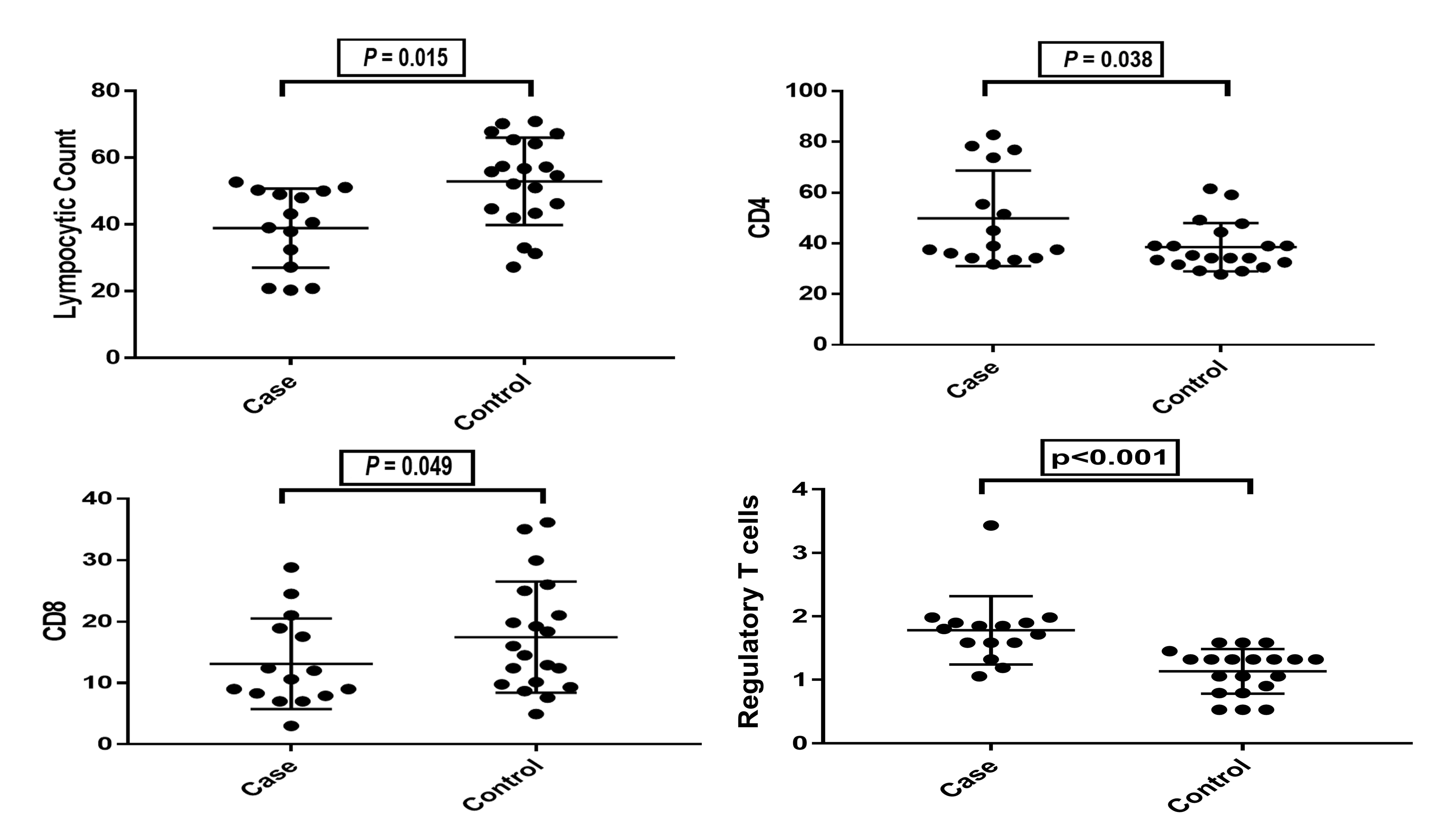

Supplement: S1 Fig — The dot graph shows a significant reduction in the percentage of total lymphocytic count in BC patients while CD4+ showed a significant increase, however there is a mild reduction in CD8+ in patient group. The percentage of Treg cells shows a significant increase in the patient group than the control (P = 0.015, P = 0.038, P = 0.049, and P < 0.001, respectively) (Grey line represents the median). (TIF) [file pntd.0011258.s001.tif]

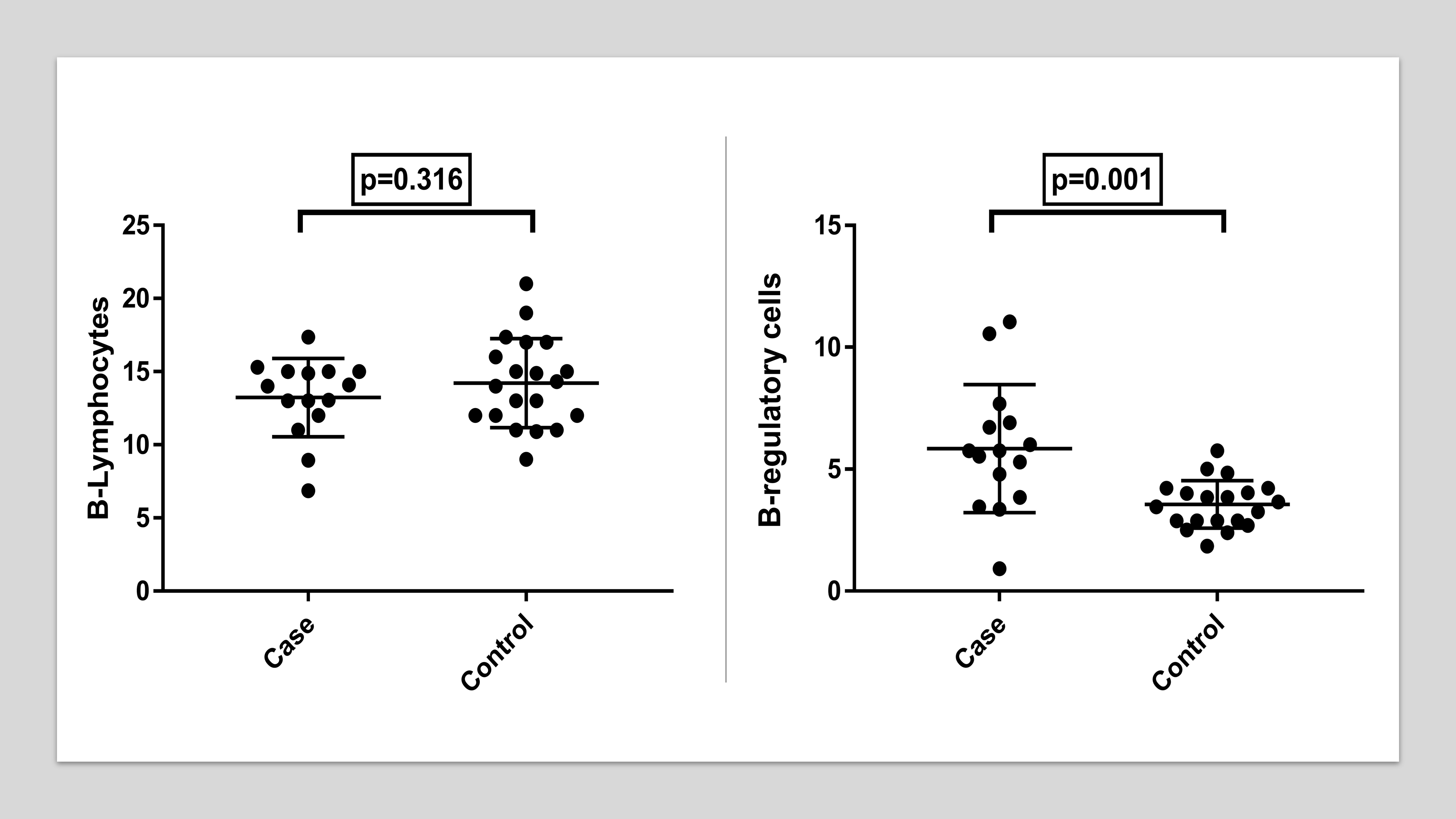

Supplement: S2 Fig — The graph shows the percentages of total B lymphocytes and Breg cells in BCa patients and control. Medians are shown as gray lines and data are compared by a non-parametric Mann-Whitney-U test. The dot graph shows a significant increase in B regulatory cells in the patient group while the total B cell number is lower in patients versus the healthy control with no statistical significance. (TIF) [file pntd.0011258.s002.tif]
